# Efficient and Reversible Electron Doping of Semiconductor-Enriched Single-Walled Carbon Nanotubes by Using Decamethylcobaltocene

**DOI:** 10.1038/s41598-017-05967-w

**Published:** 2017-07-28

**Authors:** Jian-Long Xu, Rui-Xuan Dai, Yan Xin, Yi-Lin Sun, Xian Li, Yang-Xin Yu, Lan Xiang, Dan Xie, Sui-Dong Wang, Tian-Ling Ren

**Affiliations:** 1 Institute of Microelectronics, Tsinghua National Laboratory for Information Science and Technology (TNList), Tsinghua University, Beijing, 100084 China; 2 Institute of Functional Nano & Soft Materials (FUNSOM), Jiangsu Key Laboratory for Carbon-based Functional Materials & Devices, Soochow University, Suzhou, 215123 Jiangsu Province China; 3 Laboratory of Chemical Engineering Thermodynamics, Department of Chemical Engineering, Tsinghua University, Beijing, 100084 China; 4 Department of Chemical Engineering, Tsinghua University, Beijing, 100084 China

## Abstract

Single-walled carbon nanotubes (SWCNTs) offer great potential for field-effect transistors and integrated circuit applications due to their extraordinary electrical properties. To date, as-made SWCNT transistors are usually p-type in air, and it still remains challenging for realizing n-type devices. Herein, we present efficient and reversible electron doping of semiconductor-enriched single-walled carbon nanotubes (s-SWCNTs) by firstly utilizing decamethylcobaltocene (DMC) deposited by a simple spin-coating process at room temperature as an electron donor. A n-type transistor behavior with high on current, large *I*_*on*_/*I*_*off*_ ratio and excellent uniformity is obtained by surface charge transfer from the electron donor DMC to acceptor s-SWCNTs, which is further corroborated by the Raman spectra and the ab initio simulation results. The DMC dopant molecules could be reversibly removed by immersion in N, N-Dimethylformamide solvent, indicating its reversibility and providing another way to control the carrier concentration effectively as well as selective removal of surface dopants on demand. Furthermore, the n-type behaviors including threshold voltage, on current, field-effect mobility, contact resistances, *etc*. are well controllable by adjusting the surface doping concentration. This work paves the way to explore and obtain high-performance n-type nanotubes for future complementary CMOS circuit and system applications.

## Introduction

Single-walled carbon nanotubes (SWCNTs) are promising for post-silicon nanoelectronics especially for field-effect transistor applications on rigid and flexible substrates due to their extraordinary properties including high mobility, low costs, high transparency, excellent robustness and one-dimensional structure that can provide excellent performances at scaled transistor channel lengths both at the device and system level^1–6^. To obtain the most performance benefits from scaled carbon nanotube field-effect transistors (CNFETs), circuits and systems made from carbon nanotubes must be fulfilled *via* the complementary logic types utilizing both p- and n-type transistors rather than unipolar ones because unipolar logic circuits usually sustain high steady-state currents and thus resulting in significant steady-state power consumption during operation^6, 7^. However, n-type doping of semiconductor-enriched SWCNTs (s-SWCNTs) has still remained challenging since the as-fabricated CNFETs are usually p-type in ambient due to the electron withdrawal effects by the absorbed oxygen on nanotube surface^8^. In this regard, surface charge transfer is a widely adopted and effective n-type doping technique through directly depositing electron donor materials onto nanotubes^9^. In this approach, by the choice of surface absorbed species with proper reduction potentials, the electron donation from dopants to nanotubes and thus n-doping effects of dopants on﻿ nanotubes can be induced. Notably, in comparison with conventional substitutional doping methods, the surface charge transfer method does not induce any structural defects in the lattices^10^. A range of electron donating molecules such as dihydronicotinamide adenine dinucleotide (NADH), benzyl viologens (BV), poly (ethylene imine) (PEI), hydrazine, *etc*. have been shown to be highly effective in donating electrons to nanotubes for n-type doping, which can be easily deposited by simple solution processing methods^11–15^. Moreover, to achieve robust digital circuits with high immunity against the effects of electronic noise in the electronic systems, it is important and necessary to control the threshold voltage of transistors because the threshold voltage determines the input voltage at which the circuit switches between two logic states^7^. If the threshold voltage cannot be exactly controlled during the fabrication process, the circuit might not work reliably due to the electrical noise that is always present in the electronic system^7, 16–18^. However, it still remains challenging for CNFET devices while there are only few works reported about tuning the threshold voltage of n-type CNFETs^19^.

Here we demonstrate the efficient and reversible n-type doping of s-SWCNTs by adopting decamethylcobaltocene (DMC) as an efficient surface charge transfer electron donor. DMC doping was previously reported to be applied as efficient surface charge transfer electron dopants for organic host semiconductors and novel 2-dimensional (2D) materials including pentacene, copper phthalocyanine (CuPc), fullerenes and graphene due to its extremely low solid-state ionization energy (IE) of 3.3 eV and twelve π electrons induced strong adhesion with doped material surface, demonstrating its stable electron donating characteristics and thus n-doping effects on them^20–24^. The surface charge transfer based n-doping effects is much more preferred compared with the chemically modified methods^25–28^, where the charge transfer process occurring in undoped semiconductors always needs complicated synthesis process. In this paper, we demonstrate the n-doping effects of DMC molecules on s-SWCNTs by a simple solution process. N-type CNFETs with high-performance transistor behaviors and excellent uniformity are obtained, which also show high stability, demonstrated by time-dependent electrical measurements. Electron transfer from DMC to s-SWCNTs generates an electron transfer complex, allowing for extensive charge transport characterization and practical CMOS circuits applications. A surface charge transfer model was proposed to be responsible for the above n-doping behaviors, confirmed by Raman spectroscopy an﻿d the ab initio simulations. Furthermore, the n-type transistor behaviors including threshold voltage, on current, field-effect mobility, contact resistances, *etc*. are well controllable by adjusting the DMC concentration. This work provides us a novel and effective way for fabricating high-performance n-type CNFETs, making them promising for future CMOS circuit and system applications.

## Results

### Characterization of As-Fabricated P-type CNFETs

**Figure 1:**
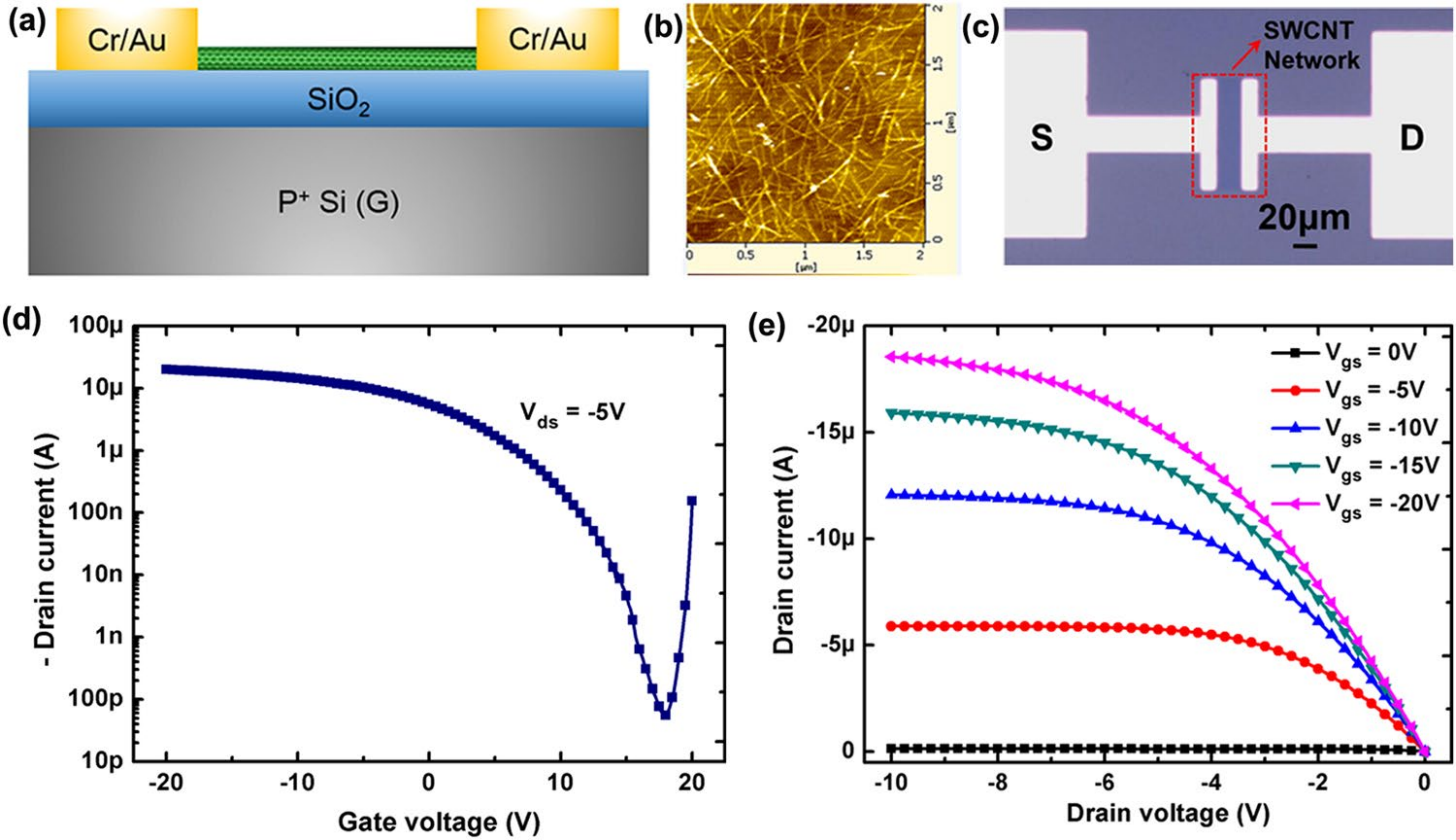
Structural and electrical characterization of back-gated CNFET devices before DMC n-doping. (**a**) The cross-sectional view of a back-gated CNFET device. (**b**) AFM image of the deposited carbon nanotube network on SiO_2_/Si substrates. (**c**) the optical image of a representative CNFET device. (**d**) Transfer *I*_*ds*_-*V*_*gs*_ and (**e**) output *I*_*ds*_-*V*_*ds*_ curves of a back-gated CNFET device which show p-channel transistor behaviors due to oxygen doping.

The effects of DMC on the electrical properties of nanotubes were firstly examined by fabricating back-gated CNFETs based on random nanotube networks (as shown in Fig. 1a). The detailed fabrication process can be seen in the Methods section. Figure 1b presents the AFM image of the obtained nanotube network on SiO_2_/Si substrates, from which a densely packed nanotube network suitable for TFT applications can be clearly seen. The channel region was defined by photolithography and then oxygen plasma etching to remove the extra nanotubes. Finally, the wafers were annealed in argon atmosphere for 30 min at 200 °C to remove the residual resist scum which may affect the following doping process. The fabricated 4-in. wafer composed of CNFETs with ranging channel widths and lengths is shown in Figure S1 and Fig. 1c presents the optical microscopy of a back-gated CNFET device with the channel length (20 μm) and width (100 μm). Figure 1c shows the transfer curves (*I*_*ds*_-*V*_*gs*_) of the as-made CNFET device (W/L = 100 μm/20 μm) before any doping process at *V*_*ds*_ = −5 V, confirming its excellent p-channel transistor behaviors with large on current (~20 μA) and high on/off current ratio (~10^5^). The p-channel characteristics can be attributed to the electron withdrawal effects by the absorbed oxygen on nanotubes in air ambient consistent with the reported results^4, 6^. Moreover, Fig. 1d presents the corresponding output characteristics (*I*_*ds*_-*V*_*ds*_) from which excellent saturation properties with less electrical noise and linear characteristics at lower *V*_*ds*_ values can also be clearly observed.

### Efficient Electron Doping Effects of DMC on s-SWCNTs

**Figure 2:**
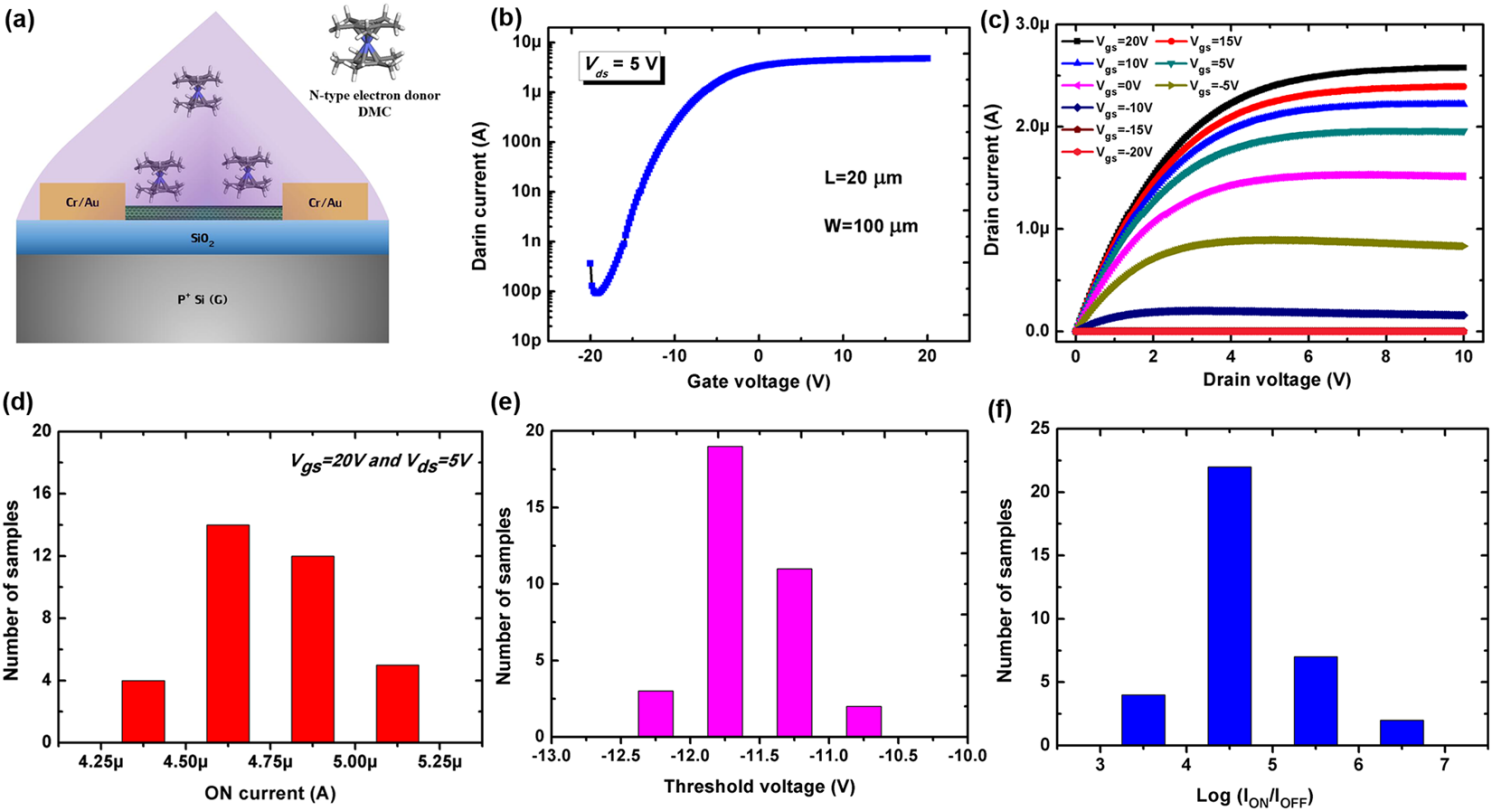
Structural and electrical characterization of back-gated CNFETs after DMC n-doping. (**a**) The schematic of the CNFET device coated with a DMC layer and the molecular structure of DMC with cobalt atom (blue), carbon atoms (gray) and hydrogen atoms (white). (**b**) Transfer curves and (**c**) output characteristics of a doped CNFET device after 5.0 wt.% with W/L = 100 μm/20 μm. Distribution of (**d**) *I*_*on*_ current at *V*_*gs*_ = 20 V and *V*_*ds*_ = 5 V, (**e**) threshold voltage and (**f**) *I*_*on*_/*I*_*off*_ current ratio of 35 n-type doped CNFET devices.

To n-dope s-SWCNTs, a DMC solution where DMC powders were dissolved in N, N-Dimethylformamide (DMF) solvent with a concentration of 5.0 wt. % was directly spin-coated onto nanotube network surface at room temperature to form a uniform doping layer. Figure 2a shows the DMC molecule structure and a schematic of a CNFET device chemically doped with DMC. As shown in Fig. 2a, a DMC molecule consists of a cobalt atom sandwiched between two methyl-substituted cyclopentadienyl groups and 19 valence electrons, and thus it has an extremely low solid-state IE of 3.3 eV, making it an optimal choice for an electron donor^20–24, 29^. For probing the doping effects, a wide range of doped CNFET devices were tested, where the prepared samples after doping process were kept in ambient air without any passivation process before, during and after electrical characterization. All the fabricated CNFET devices exhibit clear polar conversion from p-type to n-type charge transport characteristics after DMC layer deposition (the electrical transistor behaviors of all samples are recorded and counted in Fig. 2d–f) and our simple spin-coating procedure is very efficient for n-doping of nanotubes, as indicated by the transfer and output curves shown in Figs. 2b and 2c. The n-doping level is nondegenerate and still at a controllable level to allow for the excellent gate modulation effects on the nanotube channel with a high on/off current ratio of 5 × 10^4^, as presented in the transfer curves (*I*_*ds*_-*V*_*gs*_) of doped samples shown in Fig. 2b. Figure 2c presents the corresponding output curves (*I*_*ds*_-*V*_*ds*_) of the same device from which minimal electrical noise with excellent saturation and less electrical noise suitable for logic device applications can be clearly seen. Moreover, the linear behaviors of the doped device at low *V*_*ds*_ values indicate the efficient electron injection at the metal contact region, which is facilitated by DMC induced n-doping effects on nanotubes. The air-stability properties of our doped n-type devices were also studied by measuring the transfer curves of the same device immediately after the doping treatment as well as after exposure to air for 1, 2, 3, 4 and 5 weeks under a high humidity (40~80%) with no encapsulation (Figure S2). After exposure to air at such a high humidity for 5 weeks, the doped CNFET device still exhibits n-type transistor behaviors with little on current decrease and original p-type behaviors gradually appearing. However, the on/off current ratio almost keeps constant and the field-effect electron mobility first decreases and then almost remains constant after exposure in air for 3 weeks, as shown in the inset figure of Figure S2. It indicates its relatively good air-stability with slow property degradation in air, however, the stability still needs to be improved by adopting a capping layer such as CYTOP, PMMA, *etc*. in future. Moreover, the device-to-device uniformity of the DMC doping technique is very critical for exploring its possible practical applications. The transistor characteristics of 35 doped CNFET devices in one wafer were measured and their transistor parameters such as threshold voltage, on/off current ratio, on current *etc*. are extracted and counted for further analyses. Figs 2(d)–(f) present the histograms of extracted *V*_*th*_, *I*_*on*_/*I*_*off*_, *I*_*on*_ (at *V*_*gs*_ = 20 V, *V*_*ds*_ = 5 V) for our measured 35 doped CNFET devices, clearly demonstrating the uniform device-to-device distribution of our DMC n-doping technique for CNFET devices. Thus, the obtained device-to-device uniformity of doped CNFET devices is yet sufficient for the applications of this n-doping method in practical circuit and system applications such as displays, sensors, wearable electronics, *etc*.^30, 31^.

### Controllable Transistor Behaviors of n-type CNFETs by DMC doping

**Figure 3:**
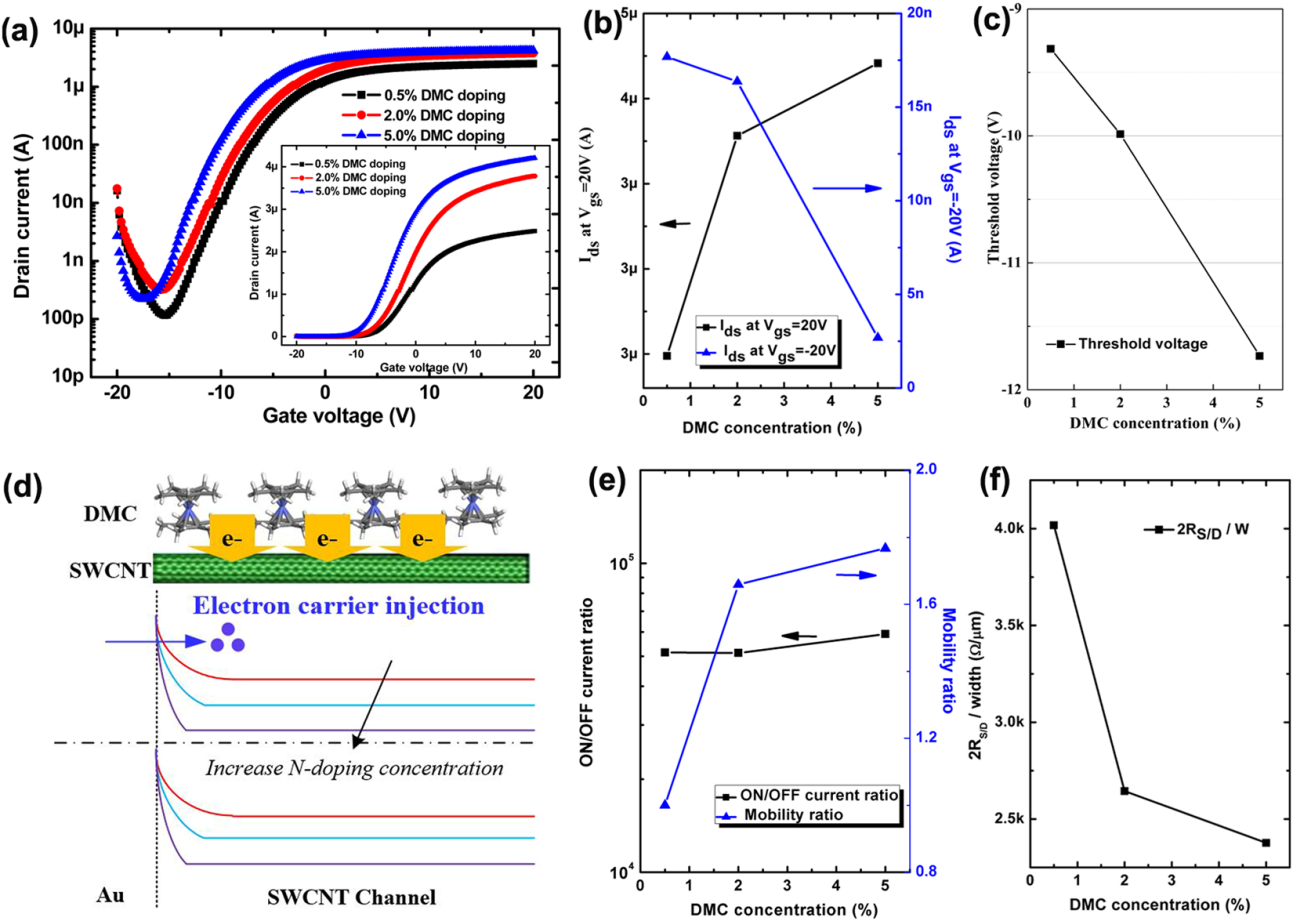
(**a**) The transfer curves (*I*_*ds*_-*V*_*gs*_) of the a CNFET device on a logarithmic scale after spin-coating DMC/DMF solutions with different concentrations ranging from 0.5 to 5.0 wt.% at *V*_*ds*_ = 5 V. Inset shows the transfer curves on a linear scale. (**b**) The drain current value (*I*_*ds*_) at *V*_*gs*_ = 20 V and −20 V as a function of DMC concentration. (**c**) Threshold voltage value of the doped CNFET device as a function of DMC concentration. (**d**) The schematic energy band diagrams of the Au-SWCNT heterojunction exhibiting the electron barrier height and region width reduced by increasing DMC concentrations. (**e**) The ON/OFF current ratio (*I*_*on*_/*I*_*off*_) at *V*_*ds*_ = 5 V and the field-effect electron mobility ratio (*μ*_*FE*_ ratio = *μ*_*FE*−*DMC*_/*μ*_*FE-DMC*=*0*.*5wt*.%_) as a function of DMC concentrations. (**f**) Contact resistance extracted by using the gated transmission line method as a function of DMC concentrations.

N-type electron donor DMC layer spin-coating is the most crucial step in this doping technique. In our n-doping process, the DMC/DMF solution was directly spin-coated onto the CNT channel region and thus the n-doping extent mainly depends on the amounts of DMC molecules deposited onto the CNT channel surface. Therefore, to further examine the n-doping effects of DMC surface functionalization on the electrical transport behaviors of CNFET devices, DMC/DMF solutions with different DMC concentrations ranging from 0.5 to 5.0 wt.% were spin-coated onto the channel surface and their electrical characteristics were then measured and analyzed. Figure 3a shows the transfer characteristics of the CNFETs (W/L = 100 μm/20 μm) after spin-coating DMC/DMF solutions with different concentrations at *V*_*ds*_ = 5 V measured in air ambient and at RT. The drain voltage applied between the drain and source electrodes was fixed at 5 V for all samples during all electrical measurements. When the DMC concentration is 0.5 wt.%, the p-type behaviors almost disappear and the doped device convert from original p-type to n-type behaviors, proving that DMC has an excellent doping level on carbon nanotubes even at such a low concentration. A progressively heavier n-doping can be observed with increasing doping concentrations, reflected by an increasing *I*_*on*_ at *V*_*GS*_ = 20 V and diminishing current at *V*_*GS*_ = −20 V, as shown in Fig. 3b. Moreover, the n-doping level is still nondegenerate and the gate control over the channel conduction is still large when the DMC concentration increases to a high doping concentration (5 wt.%), displaying the large doping tenability *via* DMC concentration. Figure 3c presents the extracted threshold voltage (*V*_*th*_) value *versus* doping concentration, from which it can be seen that the threshold voltage of doped CNFET devices was negatively shifted upon the increase of doping concentration. In our doped devices, when DMC is spin-coated onto the carbon nanotube surface, surface charge transfer occurs between DMC dopants and carbon nanotube channel layer and depletion layers of electrons and holes are present on either side of the heterojunction due to higher work function of carbon nanotubes than that of DMC. As a result, a surface dipole builds up and is spatially confined to the first molecular layers of DMC dopants and doped CNTs, resulting in the upward shift of the vacuum level and Fermi level in CNTs^32, 33^. When the doping concentration increases, more electrons will transfer from DMC to s-SWCNTs, leading to larger upward shift of the vacuum level and Fermi level and thus stronger n-doping effects. The larger upward shift of Fermi level will cause a smaller Schottky barrier width for electron injection with increase of doping concentration at Au/s-SWCNT interfaces, as shown in band diagrams (Fig. 3d). It provides an excellent and convenient way to obtain the n-type CNFET devices with the desired threshold voltage value by adjusting the doping concentration for carbon nanotube based CMOS circuits and electronic systems.

Moreover, the electron field-effect mobility also increases with the increase of doping concentration, as shown in Fig. 3e. The electron field-effect mobility increases rapidly at low doping concentration, however, it increases much more slowly at higher doping concentrations. The slower gain is likely to originate from the aggregation of dopants for high doping concentration, which limits the contact between the DMC dopant and underlying s-SWCNTs and perturbs the morphology of nanotube networks and the tube-to-tube contacts^19^. Furthermore, as presented in Fig. 3e, the on/off current ratio of the doped samples almost remains constant at 5~6 × 10^4^ for all doping concentrations. Therefore, DMC is confirmed to be an effective n-type dopant for carbon nanotubes with excellent gate control abilities, high on/off current ratio, large on current and accurate and continuous threshold voltage tuning of the n-type semiconducting behaviors by adjusting the doping concentration. In particular, as a result of the reduced electron injection barrier width, the contact resistance reduction is expected, confirmed by Fig. 3f where the contact resistance value is extracted from Fig. 3a by using the gated transmission line method^34, 35^.

## Discussions

### Reversible Doping Behaviors and Surface Charge Transfer Induced Electron Doping Mechanisms

**Figure 4:**
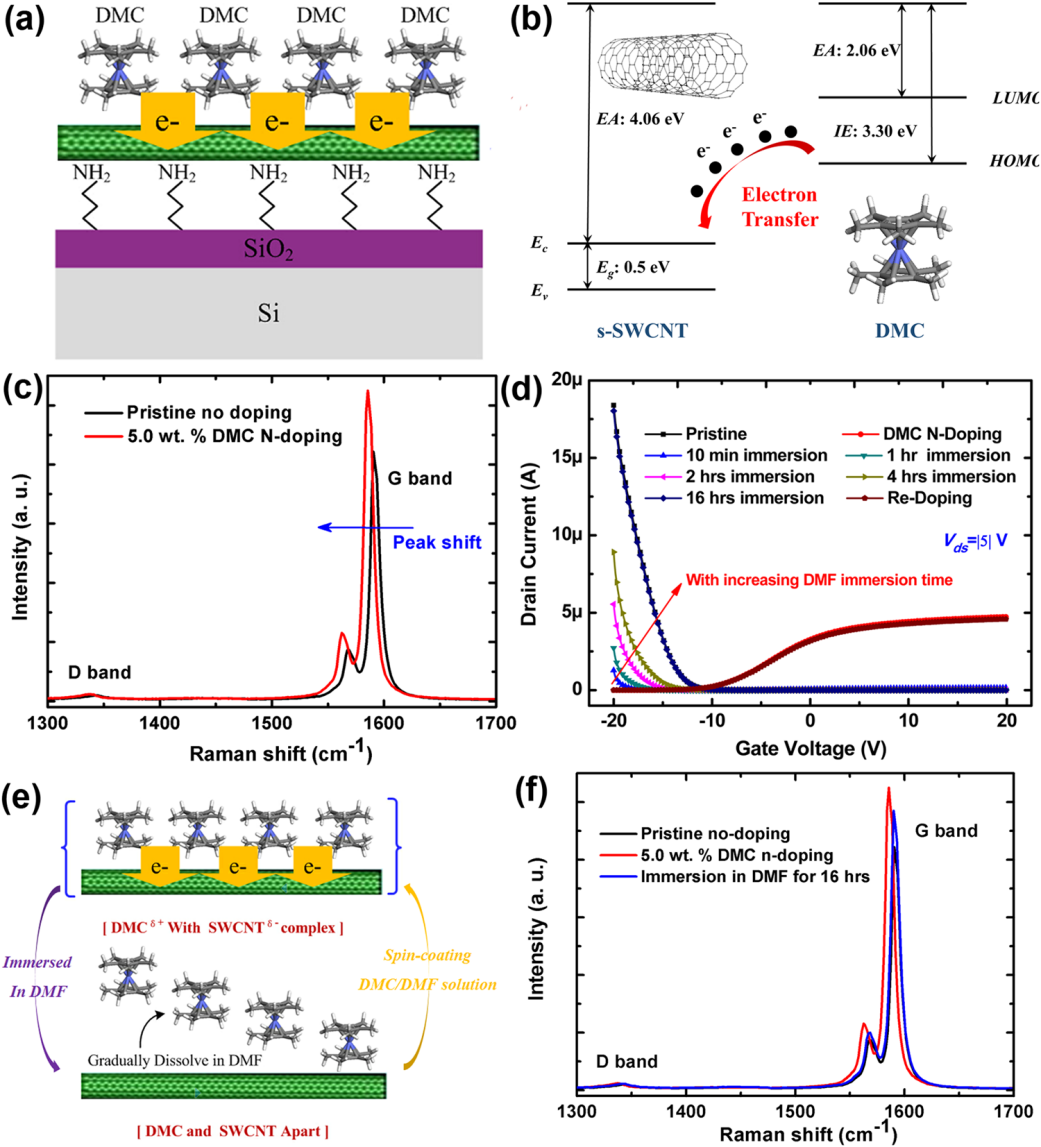
(**a**) The schematic illustration of our proposed DMC based surface charge doping process. (**b**) The energy band diagrams of s-SWCNT and DMC redox states showing the electron transfer process from higher electron energy level material (DMC) to lower materials (s-SWCNTs) which gives rise to n-type doping induced by surface charge transfer. (**c**) The Raman spectrum of pristine nanotube networks before any doping process and that after 5.0 wt. % DMC n-doping where the observed G-peak downshift indicates the effective n-type doping effects induced by surface charge transfer process. (**d**) Transfer characteristics of a CNFET device before any doping process, after DMC doping, after immersion in DMF for different periods of time and after re-doping process to depict the reversible properties of DMC n-type doping on nanotubes. (**e**) The schematic illustration of the desorption/adsorption process of DMC molecules from/onto the nanotube surface through immersion in DMF solvent or by spin-coating DMC/DMF solution, respectively. (**f**) Raman spectra of nanotubes before any doping process, after DMC doping and after immersion in DMF for 16 hours, indicating the reversibility of DMC doping process and almost no induced structural damage.

From the above electrical characterizations, it can be seen that DMC is an effective electron donor for nanotubes and results in high-performance n-type s-SWCNTs and thus n-type ﻿CNFETs. The DMC molecule is composed of a cobalt atom sandwiched between two methyl-substituted cyclopentadienyl groups and owns 12 π electrons (6 from each substituted cyclopentadienyl ring)^23, 24^, resulting in strong π-π interactions and thus excellent adhesion effects between DMC molecule and s-SWCNTs^24^. Given the strong reduction nature of DMC, a surface charge transfer based n- doping model is proposed where the neutral molecule (DMC^0^) directly transfers electrons to the unoccupied π* states of carbon nanotubes (that is to say, to reduce the nanotubes) and eventually results in strong n-type doping effects due to its low reduction potentials compared to other accepted electron-donor organic and polymer donors, as illustrated in Figure 4a. This redox reaction may generate an electron transfer complex between DMC molecules and carbon nanotubes and thus cam be represented as: DMC^0^ + s-SWCNT^0^ → DMC^δ+^ + s-SWCNT^δ−^. The corresponding energy band diagram shown in Fig. 4b depicts the expected energy level offset between the conduction band of nanotubes and the reduction potential of DMC which shows the reduction of nanotubes by DMC molecules. It has been reported that the ionization energy (IE) and electron affinity (EA) of DMC is 3.30 eV and 2.06 eV, respectively, while the EA and Fermi level of semiconducting carbon nanotubes is 4.06 eV and close to 4.56 eV, respectively^20, 21^. Due to this energy level offset, s-SWCNT acts as an electron-acceptor material, and the DMC molecule can readily transfer electrons to nanotubes as the above redox reaction, resulting in n-type semiconducting nanotubes. Raman spectroscopy was then used to confirm our above electron donating hypothesis of DMC induced n-doping effects. Figure 4c presents the Raman spectra of pristine no-doped and DMC doped nanotube networks on SiO_2_/Si substrates. It can be seen that almost no obvious D bands can be clearly observed in all samples and the G/D band intensity ratio and wavelength remain almost unchanged, indicating that no structural defects are introduced in nanotube networks after doping since the G/D band intensity ratio reflects the formation of defects^36^. Moreover, the doped s-SWCNT samples show a downshift of about 4.95 cm^−1^ in the G-peak compared with pristine nanotube sample, consistent with the spectrum of n-type nanotube networks by electron transfer^6^, suggesting the n-doping effects of DMC molecules on semiconducting nanotubes.

Uniquely, the n-type DMC dopants could be reversibly removed from the nanotube network surface by immersion the doped sample in DMF solvents. After immersion in DMF for a period of time and drying by N_2_ flow, the n-type transistor behaviors arising from DMC doping effects weakened over immersion time with the p-type behaviors gradually recovering, and finally returned to the original p-type behaviors, as shown in Fig. 4d. Here, after immersion in DMF for 10 min, the device exhibits some ambipolar transistor behaviors where the p- and n-type transistor behaviors both exist. With the increase of immersion time in DMF, the n-type current level decreases and the p-type current level increases, and eventually after immersion in DMF for 16 hours, the device almost returns back to the original state and the transistor almost behaves the same p-type behaviors with that before any doping processes, where the hole concentration increases and electron concentration decreases with increasing immersion time in DMF and finally almost reached back to the original state. After that, the DMC solution was spin-coated onto the nanotube network surface again and the device can go back to n-type, also as shown in Fig. 4d. Therefore, the DMC n-doping process on nanotube networks is reversible as indicated in the electrical characterizations. As mentioned before, the DMC molecule and s-SWCNT form an electron transfer complex (as shown in Fig. 3a) where DMC directly transfers electrons to s-SWCNTs and thus n-doping effects of DMC on nanotubes are obtained. When the doped sample was immersed in DMF, the DMC dopant molecule desorbed form the nanotube surface and dissolved in DMF, and the doped n-type CNFET devices were finally converted back to the original p-type behaviors after immersion in DMF for enough time to remove all the dopant molecules. It indicated that the n-doping process is a reversible process, as shown in Fig. 4e.

To further confirm it, besides electrical measurements, Raman spectroscopy is also adopted to examine the structural properties of nanotube networks after immersion in DMF. Fig. 4f presents the Raman spectra of nanotube networks on SiO_2_/Si substrates before doping, after DMC doping and after immersion in DMF for 16 hours, from which it can be clearly seen that immersion in DMF is not greatly detrimental to nanotube networks and can convert them back to the original states consisting of electrical and structural properties, confirmed by the above electrical and structural measurements and analyses. It is corresponding to the surface charge transfer mechanisms. This reversible doping method shown above presents a unique feature of surface charge transfer doping mechanisms compared with conventional substitution doping process. Moreover, the dopants can be selectively removed by using proper solvents and controlling the immersion time in the solvents, which in principle presents one pathway towards patterned doping where only a section of the surface dopants can be wholly or partly selectively removed by using lithographic methods and thus forming planar structures such as p-n, p-n-p, n-p-n, p-n-n^−^, p-n-n^++^, *etc*. for optoelectronic device or other electronic device applications. In such devices, we can use the reversible doping method to modulate the n-type behaviors of doped carbon nanotubes in different channel regions according to the device function requirements of the optoelectronic devices.

### Ab Initio DFT Simulations of DMC-SWCNT Complexes

**Figure 5:**
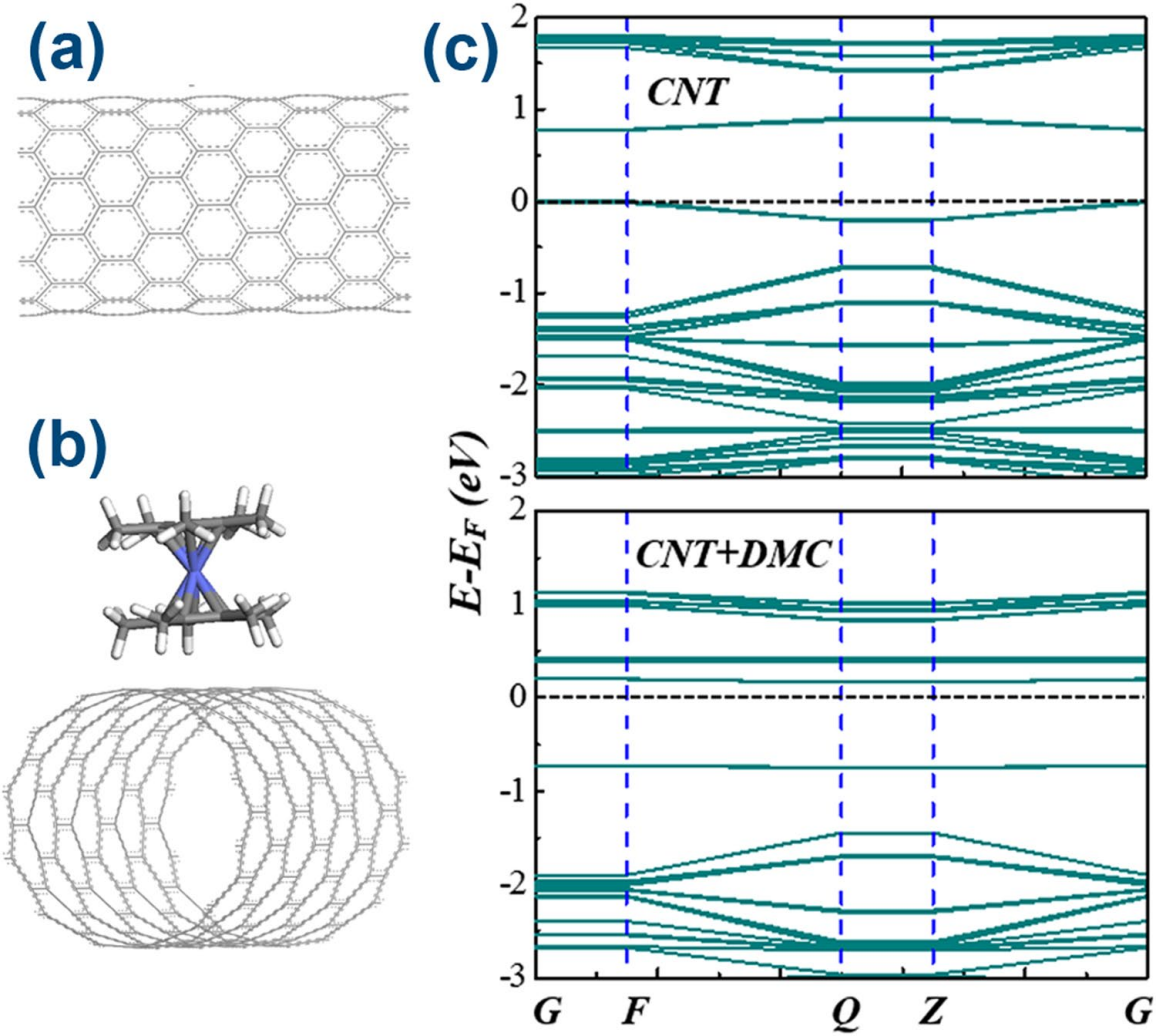
(**a**) The top view of a single-walled carbon nanotube. (**b**) The schematic view of a SWCNT-DMC system with a DMC molecule aligned to SWCNT to simulate the n-doping effects of DMC. (**c**) The electronic band structure of SWCNT before and after DMC n-doping. After DMC layer coating on nnaotubes, the Fermi-level moves away from the valence band and towards the conduction band indicating the effective n-doping effects of DMC on nanotubes.

Subsequently, ab initio density functional theory (DFT) simulations were performed to further investigate the n-doping effects of DMC molecules on s-SWCNTs. S-SWCNT with a diameter of 0.9402 nm is chosen as representative as shown in Fig. 5a. The simulation box contains an individual carbon nanotube with a single DMC molecule attached to it through van der waals (vdW) interactions. The ring of DMC parallels to the *c* axis of nanotube after the structure optimization, as shown in Fig. 5b. The band structures were calculated within the DMol^3^ package after the spin-unrestricted optimizations of both s-SWCNT and s-SWCNT-DMC systems^37, 38^. The generalized gradient-corrected Perdew-Burke-Ernzerhof (PBE-GGA) functional^39^, along with large Gaussian basis sets^40, 41^, is employed for the geometry optimization and property calculations. Here, PBE-GGA rather than BLYP functional is adopted because PBE-GGA is more suitable for long-range weak van der Waals (vdW) interactions over other GGAs while B3LYP and BLYP hybrid functional are usually used for chemical reactions^40, 42^. It is important to note that the investigation of DMC-CNT system requires careful treatment of the van der Waals (vdW) interactions between them. A dispersion correction scheme proposed by Grimme (DFT-D2) was used to describe the electronic exchange and correlation effects as well as the dispersion interactions^43^, where the incorporation of DFT-D2 scheme further improves the accuracy in evaluating weak interactions. DFT-D2 is much more accurate than DFT-D scheme and this simple approach works very well and gives reasonable results while the computation of C_6_ coeffieicent from first principles on the basis of a large database of such coefficients calculated accurately for any pair of atoms in the DFT-D3 scheme is usually complicated and time-consuming and the DFT-D3 scheme is always used to calculate the corrected energy values in a DFT-D manner^44, 45^. The 3 × 3 × 1 *k* points are sampled in the Brillouin Zone (BZ). The density mesh cutoff is 540 eV and the maximum displacement and the maximum energy is 0.005 Å and 1E-6 Ha for the geometry optimizations, respectively. The band structures (BS) of s-SWCNTs before and after DMC doping are presented in Fig. 5c. In pristine s-SWCNTs before doping, the Fermi energy level (donated by the dashed line) is located closer to the top of valence band (*E*_*V*_) compared to the bottom of conduction band (*E*_*C*_), indicating its intrinsic p-type behaviors. After doping, the Fermi energy level shifts upwards and closer to *E*_*C*_, demonstrating its n-type behaviors and the effective N-doing effects of DMC molecules on s-SWCNTs, which is consistent with the experimental results shown in Fig. 2.

In conclusion, we demonstrate an efficient and reversible n-type doping of semiconductor-enriched single-walled carbon nanotube networks with excellent transistor and uniformity performances by spin-coating DMC, an effective charge transfer donor, directly onto the nanotube network film surface. Effective and controllable n-doping levels of DMC on nanotubes and thus n-type CNFET devices have been achieved. This n-doping phenomenon is proposed to originate from the surface electron charge transfer between the dopant DMC molecule and s-SWCNTs to form an electron transfer complex, confirmed by the Raman spectroscopy and ab initio DFT calculations. The n-doped sample can convert back to the original state by immersing it into DMF and the recovered p-type behaviors can be well tuned by changing the immersion time in DMF, indicating the reversibility properties of DMC electron doping process. The n-doping levels such as on current, threshold voltage, on/off ratio, mobility, contact resistance *etc*. can be well controlled by adjusting the DMC concentrations. To be the most important, the threshold voltage of doped n-type CNFET devices can be modulated by adjusting the spin-coated DMC solution concentration where it shows an almost linear relationship *versus* DMC concentration, which is attributed to the increasing doping levels induced upward shift of the Fermi level and thus the reduced Schottky barrier width for the electron injection. This provides us a new route for high-performance obtained n-type carbon nanotube and thus n-type CNFET devices, making it a promising candidate for future carbon nanotube based electronic circuit and system applications.

## Method

### Materials

All the chemical materials were purchased and used without further purification after purchase. Poly-L-lysine (0.1% w/v in H_2_O) solution and decamethylcobaltocene were purchased from Sigma-Aldrich. N, N-Dimethylformamide (DMF) was purchased from Sinopharm Chemical Reagent Co., Ltd. (China). The 98% semiconductor-enriched suspension was supplied by NanoIntegris Inc. Deionized water used in these experiments was obtained by using a Milli-Q water system.

### Device Fabrication

Back-gated CNFET devices were fabricated on 4-in. heavily p-doped silicon substrates which also function as the back gate electrode with a thermally grown 90-nm thick SiO_2_ layer on the polished side as the gate dielectric. Photolithography was firstly adopted to pattern the source/drain (S/D) electrodes, followed by e-beam evaporation of Cr/Au (10/50 nm) and the following lift-off process to form S/D electrodes. Then Poly-L-lysine solution was drop-cast onto the SiO_2_ surface for 5 min to functionalize the SiO_2_ surface for nanotube network film deposition, followed by immersing the substrate in 98% semiconductor-enriched SWCNT suspension (NanoIntegris Inc.) for 1 hour and a following rinse with DI water and isopropanol to remove the excess solution. The channel region was defined also by photolithography and then oxygen plasma etching to remove the extra nanotubes. Finally, the wafers were annealed in argon atmosphere for 30 min at 200 °C to remove the residual resist scum which may affect the following doping process.

### DMC n-doping process

DMC with different concentrations ranging from 0.5 wt.% to 5 wt.% was firstly totally dissolved into DMF without any residual at room temperature. Then, for n-type doping, DMC solution (different concentrations ranging from 0.5 wt.% to 5 wt.% in DMF) was directly spin-coated onto the wafers at room temperature in air ambient. After spin-coating, the sample was then annealed on a hotplate at 100 °C for 10 min.

### Structural Characterization

In order to examine the surface morphologies of the as-prepared nanotube network films, the morphology of the SWCNT network films on SiO_2_/Si substrates were observed by atomic force microscopy (AFM, SPA 500, Seiko Instruments Inc.) with taping mode. All Raman spectra for undoped, DMC n-doped nanotubes and DMF washed nanotubes were excited with the 514.5 nm line of a He-Ne laser and recorded using a Renishaw Invia Raman Microscope.

### Electrical Characterization

All electrical characteristics of all fabricated CNFET devices in this paper were measured with an Agilent B1500A semiconductor parameter analyzer in ambient environment at room temperature.

The threshold voltage (*V*_*TH*_) was extracted by finding the x-intercept points of the tangential lines in the linear region of ${I}_{DS}^{0.5}$ *versus V*_*GS*_ curves (Figure S3). The field-effect mobility (*μ*_*FE*_) was calculated from the following equation:

1 $${\mu }_{FE}=\frac{L}{W{V}_{DS}{C}_{OX}}\cdot \frac{\partial {I}_{DS}}{\partial {V}_{GS}}$$

where *L* and *W* are the length and width of the channel, *V*_*DS*_ is the drain-source voltage and *C*_*OV*_ is the gate oxide capacitance per unit area.

In the linear regime, the overall device resistance (*Ron*) can be expressed as the sum of the channel resistance (*r*_*ch*_ *L*) and contact resistance (*2R*_*S*/*D*_) according to

2 $${R}_{on}={r}_{ch}L+2{R}_{S/D}=\frac{L}{W{\mu }_{FE}{C}_{G}({V}_{GS}-{V}_{TH})}+2{R}_{S/D}$$

where *μ*_*FE*_ and *V*_*TH*_ are the field-effect mobility and threshold voltage, respectively. Thus the contact resistance *2R*_*S*/*D*_ can be extracted from the above section.

## Electronic supplementary material

Supplementary Information
