# Supplementary material for: Efficient and Reversible Electron Doping of Semiconductor-Enriched Single-Walled Carbon Nanotubes by Using Decamethylcobaltocene
Source: Sci Rep. 2017 Jul 28;7:6751. doi: 10.1038/s41598-017-05967-w (PMC5533747; doi:10.1038/s41598-017-05967-w)
Supplement: Supplementary file 1 — Supplementary Information [file 41598_2017_5967_MOESM1_ESM.doc]

### Supporting Information

### Efficient and Reversible Electron Doping of Semiconductor- Enriched Single-walled Carbon Nanotubes by Using Decamethylcobaltocene

*Jian-Long Xu^1, 2*^, Rui-Xuan Dai^1^, Yan Xin^3^, Yi-Lin Sun^1^, Xian Li^1^, Yang-Xin Yu^3^, Lan Xiang^4^, Dan Xie^1*^, Sui-Dong Wang^2^, and Tian-Ling Ren^1*^*

^1^Institute of Microelectronics, Tsinghua National Laboratory for Information Science and Technology (TNList), Tsinghua University, Beijing 100084, People’s Republic of China

^2^Institute of Functional Nano and Soft Materials (FUNSOM), Jiangsu Key Laboratory for Carbon-based Functional Materials and Devices, Soochow University, Suzhou 215123, Jiangsu Province, China

^3^Laboratory of Chemical Engineering Thermodynamics, Department of Chemical Engineering, Tsinghua University, Beijing 100084, China

^4^Department of Chemical Engineering, Tsinghua University, Beijing 100084, China

Address all correspondence to the author. *Email: [RenTL@tsinghua.edu.cn](mailto:RenTL@tsinghua.edu.cn); [xujianlong@suda.edu.cn](mailto:xujianlong@suda.edu.cn); xiedan@tsinghua.edu.cn


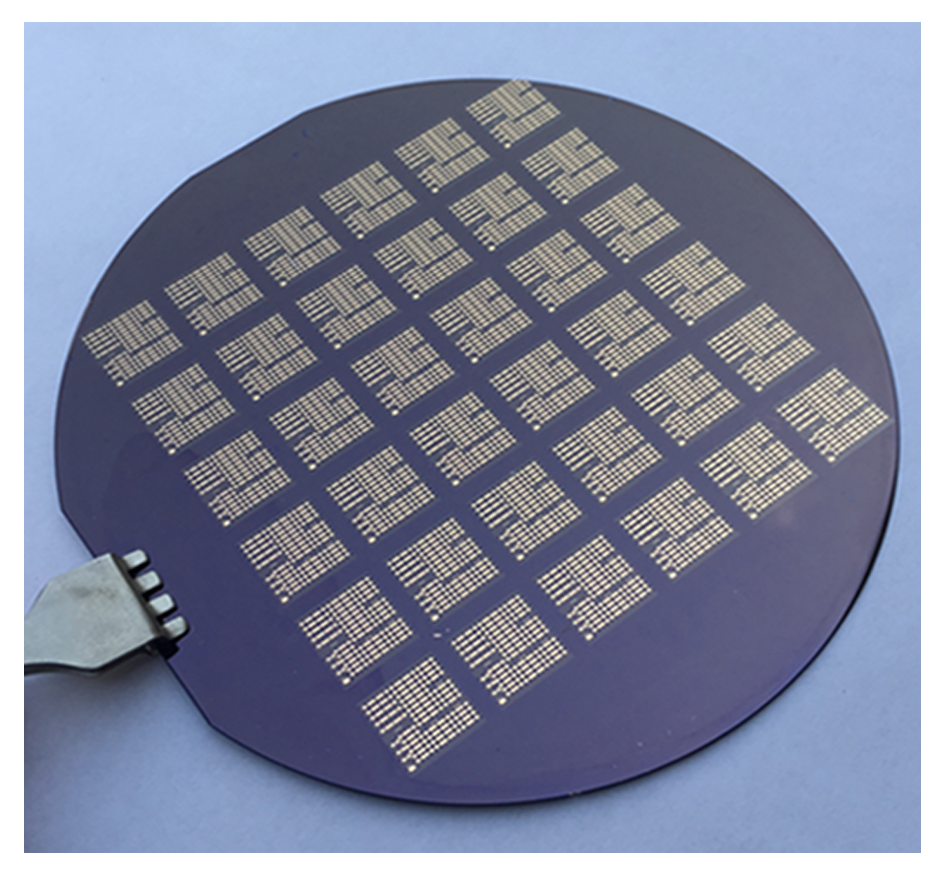


Figure S1 Fabricated 4-inch wafer with back-gated s-SWCNT TFT devices.


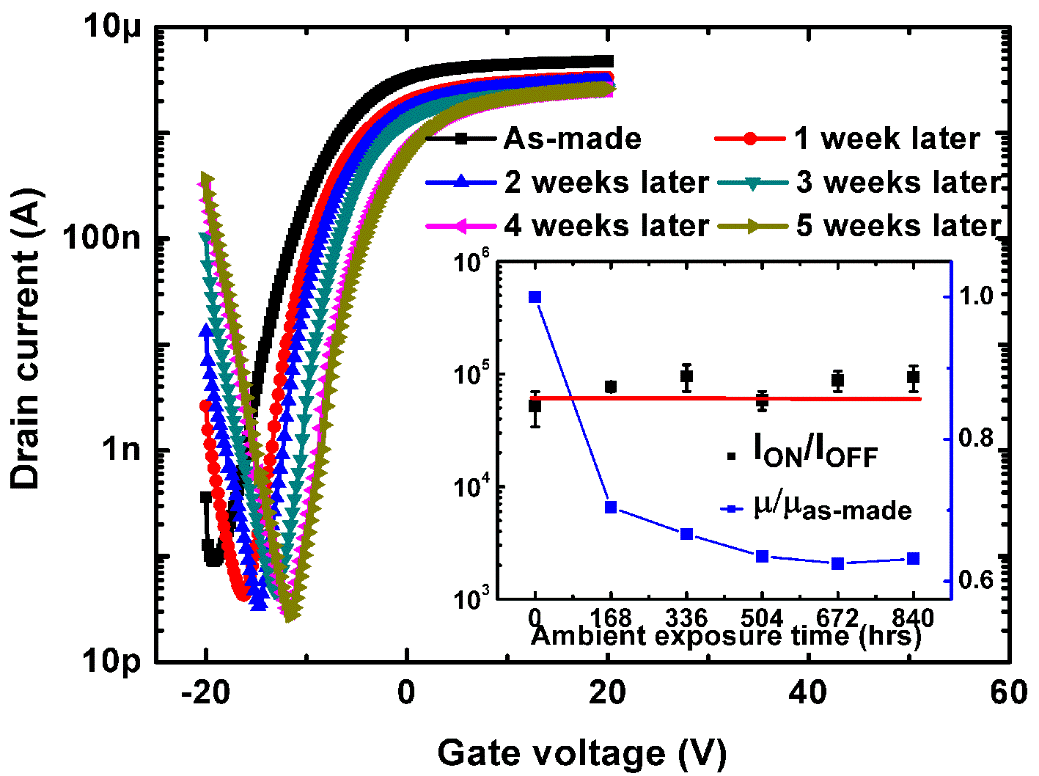


Figure S2 Transfer curves of DMC doped TFT devices immediately after DMC n-doping and after direct exposure to air for 1, 2, 3, 4 and 5 weeks without any encapsulation treatments. Inset shows the mobility and on/off current *versus* time curves


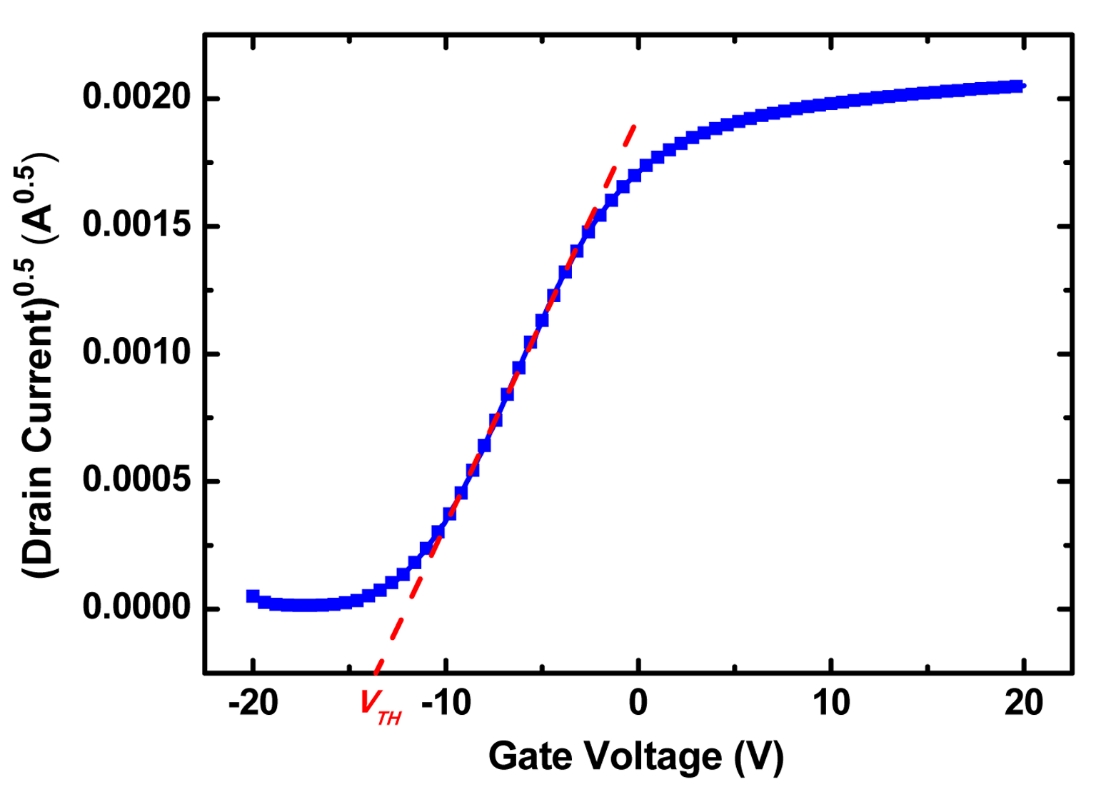


Figure S3 Determination of threshold voltage (*V_TH_*) in *I_DS_^0.5^*-*V_GS_* curves
